# Acute Exposure to Zearalenone Disturbs Intestinal Homeostasis by Modulating the Wnt/β-Catenin Signaling Pathway

**DOI:** 10.3390/toxins12020113

**Published:** 2020-02-11

**Authors:** Tarek Lahjouji, Aurora Bertaccini, Manon Neves, Sylvie Puel, Isabelle P. Oswald, Laura Soler

**Affiliations:** Toxalim (Research Centre in Food Toxicology), University of Toulouse, INRAE, ENVT, INP-Purpan, UPS, 31027 Toulouse, France; Tarek.lahjouji@inrae.fr (T.L.); bertacciniaurora@gmail.com (A.B.); manon.neves@inrae.fr (M.N.); sylvie.puel@inrae.fr (S.P.); laura.soler-vasco@inrae.fr (L.S.)

**Keywords:** mycotoxins, zearalenone, intestine, pig, Wnt/β-catenin, estrogen

## Abstract

The mycotoxin zearalenone (ZEN), which frequently contaminates cereal-based human food and animal feed, is known to have an estrogenic effect. The biological response associated with exposure to ZEN has rarely been reported in organs other than the reproductive system. In the intestine, several studies suggested that ZEN might stimulate molecular changes related to the activation of early carcinogenesis, but the molecular mechanisms behind these events are not yet known. In this study, we investigated gene expression and changes in protein abundance induced by acute exposure to ZEN in the jejunum of castrated male pigs using an explant model. Our results indicate that ZEN induces the accumulation of ERα but not ERβ, modulates Wnt/β-catenin and TGF-β signaling pathways, and induces molecular changes linked with energy sensing and the antimicrobial activity without inducing inflammation. Our results confirm that the intestine is a target for ZEN, inducing changes that promote cellular proliferation and could contribute to the onset of intestinal pathologies.

## 1. Introduction

Zearalenone (ZEN) is a mycotoxin synthesized by some fungi of the *Fusarium* genus that frequently contaminate crops such as corn, barley, wheat, rice, oats, and sorghum, particularly in temperate regions [1]. ZEN is classified as an endocrine disruptor because it can bind and activate estrogen receptors with hyper-estrogenic effects [2]. Like other endocrine disruptors, ZEN has a dual toxicity: pro-apoptotic effects at high, acute doses, and anabolic effects at low doses during chronic exposure [3]. At the cellular level, the toxic effect of high concentrations of ZEN is independent of estrogen signaling, and is characterized by a high oxidative state that induces apoptosis [3]. Indeed, ZEN provokes a dose-dependent increase of reactive oxygen species levels, oxidative DNA damage, alteration of the mitochondria, membrane disruption and lipid peroxidation [4,5,6,7]. These processes can activate a pro-inflammatory cascade [8,9], resulting in the expression of inflammatory cytokines, which has been reported in several tissues exposed to ZEN [9,10,11]. At lower doses, the in vitro toxic effect of ZEN is more dependent on the activation of the estrogen receptor (ER) signaling pathway, which has an anti-inflammatory [8,11,12], anti-apoptotic and proliferating effect [3,13,14]. ER is a ligand-activated transcriptional factor, and signaling is mainly activated directly upon DNA binding in the estrogen response elements located in target genes, but also indirectly (not involving DNA binding) through interaction with other signaling pathways [15,16,17]. There are two types of ERs, ERα and ERβ, whose roles differ. ERα is the main regulator of estrogen-dependent genes and its activation has proliferative effects. ERβ, when co-expressed with ERα, tends to restrain ERα activity and its activation inhibits cell proliferation [18]. ZEN can activate both ERs but is a partial agonist of ERβ and a full agonist of ERα [19]. Consequently, the biological response to exposure to ZEN varies depending on the tissue-specific ratio of the ER α vs. β, as well as on the density of these receptors [20]. In the intestine, the presence of each ER is distributed differently along the crypt–villus axis, ERα being more abundant in the crypt (where cell proliferation occurs) while ERβ is more abundant in the villi (composed of differentiated enterocytes) [21,22]. Estrogen signaling interacts with other pathways that are important for intestinal homeostasis and its disruption seems to relate to the development of chronic intestinal diseases and cancer [23,24,25]. As the intestine is an estrogen-responding organ, it is important to understand the molecular effect of natural endocrine disruptors such as ZEN. Moreover, due to its ability to induce both cell proliferation and oxidative DNA damage, a link has been suggested between ZEN and cancer promotion in the literature [3].

The toxic effect of ZEN in the intestine has been investigated in the past, mostly using pig as model for humans due to their similar sensitivity and toxicity [26]. The main reason for ZEN sensitivity is that pigs, similar to humans, convert ZEN into the more estrogenically active α-zearalenol [27]. In vivo, whereas some authors found no morphologic changes [28,29], others found that chronic exposure of pigs to ZEN led to transient morphological modifications in the small intestine during the first weeks of exposure [29], or to an increase in the number of Paneth cells at the bottom of the intestinal crypts [28]. At the cellular and molecular level, several reports have described changes in the gene expression of pro-inflammatory cytokines, genes implicated in the induction of a proliferative state such as Dickkopf-related protein 1 (DKK1), β-catenin or the proto-oncogene c-Myc [8,12,30]. Taken together, these results suggest that ZEN can induce transient proliferation in the small intestinal crypt, which could be connected with pro-cancerogenic changes in wingless-type MMTV integration site family (Wnt)/β-catenin and transforming growth factor β (TGF-β) signaling pathways. The two latter pathways are known to play a key role in the toxic effect of ZEN in ovarian [31], uterine [31] and prostatic cancer cells [32], and to be implicated in the onset and progression of intestinal proliferative/cancerous events [33], but the activation of these pathways by ZEN has not been investigated to date. 

In this study, we investigated the activation/repression of Wnt/β-catenin and TGF-β signaling pathways, the immune status and metabolism by ZEN to understand the effect of this toxin on the intestine of castrated male pigs. Our results confirmed that the small intestine is a target for ZEN, and that its toxic effect could contribute to the development or aggravation of severe intestinal pathologies such as cancer.

## 2. Results

**Figure 1:**
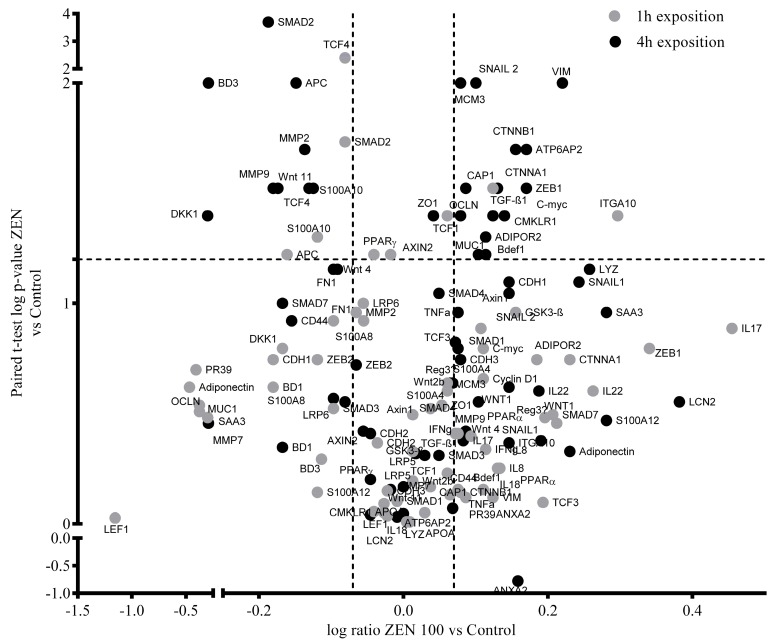
Volcano plot of log significance (paired t-tests) versus log ratio on the y and x axes of ZEN-induced changes (100 μM) in gene expression. Black and grey dots represent gene expression changes after 4 h at 1 h of exposure, respectively. Horizontal and vertical dotted lines indicate the established levels of significance (*p* < 0.05; ratio = 1.2).

The abundance of 68 gene transcripts and 14 proteins were evaluated using RT-qPCR and western blot, respectively, in pig jejunal explants from castrated male pigs exposed for 1 h or 4 h to 100 µM ZEN. Differences in gene expression were found for six transcripts after 1-h treatment and 22 transcripts after 4 h of exposure (*p* < 0.05; Figure 1; Supplementary Table S1). 

**Figure 2:**
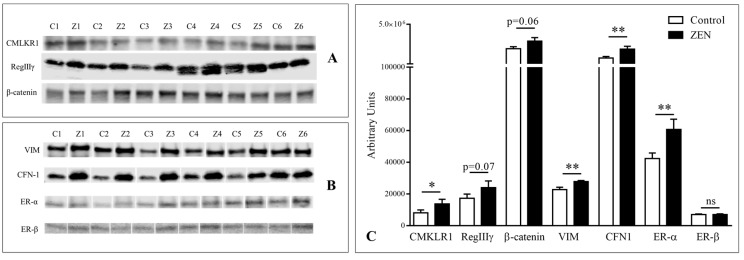
Immunoblotting analysis of ERα and ERβ, as well as proteins showing either significant of almost significant differences between six paired pig jejunal explants (1-6) exposed (Z) or not (C) to 100 μM ZEN (Z). (**A**) CMLKR1, RegIIIγ, β-catenin, (**B**) VIM, CFN-1, ERα and ERβ. (**B**) Relative quantification of normalized signal (arbitrary units). Values are means with standard errors of the mean represented by vertical bars (n = 6). Asterisks indicate statistical differences (*p < 0.05; ** *p* < 0.01).

Regarding protein expression, changes in relative abundance were significant for estrogen receptor alpha (ERα), cofilin-1 (CFL1), β-catenin, vimentin (VIM), and chemerin receptor (CMKLR1) at 4 h (*p* < 0.05; Figure 2). Zymography analysis revealed a significant decrease in the gelatinase activity of pro- and active metalloproteinase (MMP) proteins 2 (MMP2) and active metalloproteinase protein 9 (MMP9) at 4 h of exposure. A description of the results is given on the following paragraphs, and is organized according to the main signaling pathway studied.

### 2.1. Effect of Zearalenone on ER Abundance

Relative protein quantification by western blot showed a significant up-accumulation of ERα after 4 h of exposure of ZEN (*p* < 0.01), while ERβ abundance did not change at any time-point. 

### 2.2. Effect of ZEN on the Wnt/β-catenin Signaling Pathway

We investigated the effect of ZEN on the activation of the Wnt/β-catenin signaling pathway. Extracellular Wnts activate responding cells by binding to its membrane receptors Frizzeld and Low-density lipoprotein receptor-related protein (LRP). This leads to a cascade of events that allows the stabilization of cytoplasmic β-catenin and its translocation of the nucleus through a process that depends on the activity of glycogen synthase kinase 3 beta (GSK3β) and is inhibited by the proteins adenomatous polyposis coli (APC) and axins. In the nucleus, β-catenin associates with T-cell factor/lymphoid enhancer-binding transcription factors (TCF/LEF) to induce the expression of target genes, such as Cyclin 1, Zinc Finger E-Box Binding Homeobox 1 and 2 (Zeb1/2), Snail1, Snail2, and c-Myc among others. The whole process is inhibited by DKK-1 [34].

Several genes of this signaling cascade were up-regulated by 4 h of exposure to ZEN, namely β-catenin (*p* < 0.05; showing a similar trend at the protein level, although the up-accumulation was only almost significant; *p* = 0.06), Cyclin D1 (*p* < 0.05), c-Myc (*p* < 0.05), Zeb1 (*p* < 0.05) and Snail2 (*p* < 0.01), whereas TCF1 was up-regulated at 1 h of exposure (*p* < 0.05). A significant down-regulation was observed at 1 h for TCF4 (*p* < 0.01), and at 4 h (*p* < 0.05) for Wnt11 (*p* < 0.05), DKK1 (*p* < 0.05), APC (*p* < 0.01). Nearly significant down-regulation of axin2 (*p* = 0.06) and APC (*p* = 0.06) at 1 h was also observed. The gene expression of ATPase H+ transporting accessory protein 2 (ATP6ap2), a facilitation of the transmission of β-catenin signaling [35], was significantly induced at 4 h (*p* < 0.05). 

To link the activation of Wnt/β-catenin with cell proliferation, we investigated two markers of cell proliferation, minichromosome maintenance complex component 3 (MCM3; *p* < 0.05) and Vimentin (VIM; *p* < 0.01), whose expression was significantly up-regulated at 4 h of exposure. VIM was also significantly up-accumulated at 4 h of exposure at the protein level (*p* < 0.001).

Among the expected consequences of β-catenin transcriptional activation is the repression of the expression of the tight junctional proteins Zonula occludens-1 (ZO-1), claudins, occludins as well as the adherens junctional proteins cadherins and α-catenin [36]. No changes in cadherins were observed at the transcriptional or protein level at any time-point. Significant up-regulation of occludin (*p* < 0.05), ZO-1 (*p* < 0.05) and α-catenin (*p* < 0.05) gene expression was observed at 4 h of exposure. No differences in occludin and ZO-1 were observed at the protein level, whereas significant up-accumulation of the actin cytoskeleton protein CFL1 was found at 4 h of exposure. (*p* < 0.05).

Paneth cells are small-intestine epithelial cells located at the bottom of the crypts. They contain secretory granules containing antimicrobial proteins and peptides, but also play a role in regulating proliferation in the crypt. As their quantity and maturation depends on Wnt/β-catenin signaling [37,38], we investigated the abundance of several antimicrobial peptides. At 4 h of exposure., we observed an almost significant over-expression of β-defensin 1 (*p*BD1; *p* = 0.05), down-regulation of the expression of β-defensin 3 (*p*BD3; *p* < 0.05) together with a nearly significant over-expression of lysozyme at the transcript level (*p* = 0.06). The abundance of regenerating islet-derived protein 3 gamma (RegIIIγ) was investigated by western blot, and an accumulation was observed, although it was only nearly significant (*p* = 0.07) at 4 h.

Taken together, our results provide evidence that ZEN can activate the Wnt/β-catenin signaling pathway without affecting the intestinal barrier, and induce the expression of proliferation biomarkers as well as antimicrobial proteins.

### 2.3. Effect of ZEN on the TGF-β-catenin Signaling Pathway

In the intestine, the Wnt signaling pathway interplays with other pathways, including the TGF-β signaling pathway. TGF-β signaling is mediated through transmembrane receptors that use Smad2 and Smad3 proteins to transduce their signals from the cell surface to the nucleus in a process that requires the presence of Smad4 for DNA binding and transcriptional activation of target genes, and is inhibited by Smad 7 [24,39]. 

**Figure 3:**
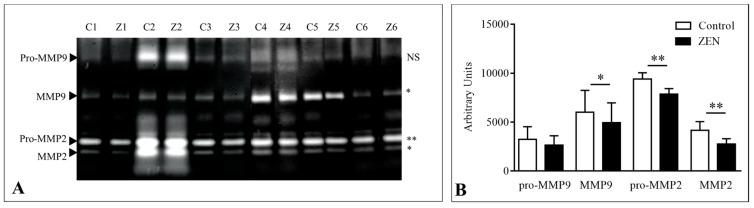
(**A**) Gelatinolytic activities of jejunal explant culture medium from the control (C) and at 4 h of exposure to ZEN (Z) paired samples from six pigs (1–6). Arrows indicate the position of pro-MMP9, active MMP9, pro-MMP2, and active MMP2. (**B**) Relative quantification of gelatinolytic activities. The level of significance of paired *t*-test analysis of the differences in gelatinolytic activity is given on the right for each band (* *p* < 0.05; ** *p* < 0.01; NS: not significant).

Transcript levels of TGF-β1 were significantly up-regulated at 1 h of exposure (*p* < 0.05), but no difference was found at 4 h. The expression of all the Smads studied (1,2,3,4 and 7) remained unchanged at all time points, with the exception of Smad2, whose transcript levels were significantly down-regulated at 1 h (*p* < 0.05) and 4 h of exposure (*p* < 0.001). We investigated several target genes in this pathway, and found significant up-regulation of Integrin-alpha 10 at 1 h (*p* < 0.05), no changes in the expression of the CD44 molecule (Indian Blood Group; CD44) nor MMP7, and down-regulation of the expression of fibronectin (nearly significant; *p* = 0.07), as well as MMP2 and MMP9 (*p* < 0.05) at 4 h of exposure. Gelatinase activity of MMPs leaked to the explant medium was analyzed and whereas no differences were found at 1 h of exposure nor pro-MMP9 at 4 h, the intensity of bands decreased significantly at 4 h of exposure to ZEN for active MMP9 (*p* < 0.05), pro-MMP2 (*p* < 0.01) and active MMP2 (*p* < 0.01; Figure 3). 

These results provide no evidence for ZEN-dependent activation of the TGF-β signaling pathway.

### 2.4. Effect of Zearalenone on Cytokine, Inflammatory Markers, and Adipokine Expression

To investigate if ZEN can induce intestinal inflammation under our conditions, several cytokines and acute phase proteins were analyzed. No differences were found in the expression or protein abundance of all the inflammatory markers investigated, namely interleukins 8, 17, 18, 22, tumor necrosis factor α (TNF-α) and interferon gamma (IFNγ), protein S100A8, protein S100A12, serum amyloid A3 (SAA3), alpha-acid glycoprotein alpha 1 (α-1 AGP) and lipocalin 2 (LCN2). Regarding the complex S100A10/AnnexinA2 (ANXA2), no changes were observed in the gene expression of ANXA2, whereas a marked down-regulation of was significant at 4 h of exposure (*p* < 0.05), and almost significant at 1 h (*p* < 0.06), of protein S100A10.

We investigated the expression of peroxisome proliferator activated receptors (PPARs), since ZEN exposure can increase energy production through an increase in the uptake and β-oxidation of fatty acids, in a processes that has been suggested to be mediated by activation of PPARs in Leydig cells [40]. However, we observed no changes in PPARα or in PPARγ gene expression at any time-point. The expression of several adipokines and their receptors was also investigated to compare it with previous results that produced evidence for regulation on the expression of the chemerin receptor [30]. No differences were found in the expression of adiponectin, whereas the mRNA levels of the adipokines receptors for adiponectin (Adiponectin receptor 2; AdipoR2), resistin (cyclase associated actin cytoskeleton regulatory protein 1; CAP1) and chemerin (chemerin chemokine-like receptor 1; CMKLR1, confirmed at the protein level) were significantly up-regulated at 4 h of exposure (*p* < 0.05).

These results show that no inflammation was associated with the treatment with ZEN, whereas significant changes were associated with adipokine receptors.

## 3. Discussion

Estrogen signaling is important for intestinal homeostasis, but disturbance of the mycoestrogen ZEN in the intestinal molecular pathways that interact with that of estrogen, such as Wnt/β-catenin and TGF-β, is not completely understood. Unfortunately, most of the molecular pathways that regulate intestinal physiology cannot be studied in vitro, since they are compartmentalized or present as a gradient that can increase or decrease along the crypt–villus axis. Here, an ex-vivo model of jejunal explants was used to reflect the complexity of the interaction between the different cell types present in the intestine. The pig is a ZEN-sensitive species [26], and resemblances in anatomy, physiology and genetics makes pig the best model for human intestinal studies [41]. Moreover, the use of control-treated paired explants allowed us to account for individual variations and hence to detect the subtle changes associated with the toxicity of ZEN. Given that the exposure time in this model is restricted to a maximum of 4 h, we used a high dose of toxin that would induce as many molecular changes as possible, while that the short exposure time ensures that cell mortality is low enough so analysis will not be polluted by unspecific events [42]. As the present study aims at the study of the ZEN toxicity in intestinal explants for the first time, we employed castrated immature males in order to avoid the known influence of the animal hormonal status of exposed animals in ZEN toxicity [43].

The small intestinal mucosa is a barrier against food contaminants. It is formed by an epithelial cell lining that is continuously renewed in a process that depends on the division of intestinal stem cells (ISCs), located near the base of the crypt [33,44]. Proliferation is governed mainly by the Wnt/β-catenin pathway that is present in an increasing gradient from the base to the bottom of the crypts, whereas TGF-β superfamily proteins (including BMPs, GDFs) maintain a state of growth equilibrium and promote epithelial differentiation towards the tips of the villus [34]. ER activity regulates the Wnt/β-catenin and TGF-β pathways [21,22]. ZEN is known to alter the balance between ER, Wnt/β-catenin and TGF-β signaling in prostatic cancer cells as well as ovarian cells, these being linked with the pro-proliferative and cancerogenic effects of ZEN [31,32]. Here, we demonstrate that ZEN induces an accumulation of ERα, but no ERβ, in the intestine, and modulates the Wnt/β-Catenin and TGFβ signaling pathways in the intestine.

Under our conditions, significant accumulation of ERα was observed, whereas the levels of ERβ remained unchanged. The accumulation of ERα can explain other molecular changes observed in the present study such as the activation of the Wnt/β-catenin signaling pathway [15,16,17]. Significant down-regulation of the inhibiting factors DKK1 and APC was observed, together with up-regulation of β-catenin (with a nearly significant protein up-accumulation), Cyclin D1, c-Myc, Zeb1, and Snail2. We observed down-regulation of the transcription factor TCF4, which seemed to conflict with the fact that TCF4 is major transcriptional mediator of the Wnt signaling pathway. In contrast, we observed up-regulation of the expression of TCF1 after 1 h of exposure. In mice, the epithelial expression of TCF4 is indispensable for cell proliferation and tumor initiation but in humans, the TCF4 role is redundant with the related TCF1 and LEF1 transcription factors [45]. No information is available on how signaling is regulated in pig, but up-regulation of the most important target genes, such as c-Myc and Cyclin D1, confirms that down-regulation of TCF4 is not essential for β-catenin-mediated transcriptional activation. We also observed significant up-regulation of the expression of ATP6AP2, a protein that is necessary for the facilitation of the transmission of Wnt/β-catenin signaling [35], and whose ZEN-dependent up-regulation has already been observed in the pig duodenum [12]. Our results suggest that the activation of Wnt signaling could be mediated by the up-accumulated ERα, since it is known that ERα down-regulates the Wnt inhibitor DKK1. The ability of ZEN to suppress DKK1 in intestinal tissues has been already described; in that study, the ability was linked with possible modulation of epithelial growth factor receptor (EGFR) signaling, which is closely connected with Wnt signaling [30]. ERα can also activate Akt1, which inhibits GSK-3β and favors β-catenin nuclear translocation and subsequent transcription of Wnt target genes [23,46]. The activation of Akt1 by ZEN has also been described in other tissues including granulosa [47], Sertoli [48] and Leydig cells [49]. Among the Wnt transcripts we investigated, we only observed a change in Wnt11. Wnt11 might function as a tumor suppressive gene that can inhibit Wnt signaling [50,51]. According to our results and those of and other authors, ZEN effectively inhibits the Wnt repression system through repression of DKK1. It would be interesting to investigate if Wnt11 is also down-regulated by ZEN to act as a Wnt repressor. The activation of the pro-proliferative Wnt/β-catenin pathway was further confirmed by the significant up-regulation of proliferation biomarkers MCM3, and VIM, confirmed at the protein level for the two latter markers. Results additionally indicate that ZEN does not remodel tight nor adherens junctions but is able to promote cytoskeletal changes related to enhanced migration, such as the regulation of the expression of α-catenin and CFN-1. 

There might be a link between the ZEN-induced activation of Wnt/β-catenin signaling and the detected changes in antimicrobial peptides. Although our results were not significant, up-regulation/up-accumulation of the antimicrobial proteins lysozyme and RegIIIγ produced by the Paneth cells was observed. Previous reports report a ZEN-induced increase in Paneth cells in pig [28]. The homeostasis of Paneth cells depends on ER [21] and Wnt/β-catenin [37], and dysregulation in their function is related to the development of cancer [22] and chronic inflammatory diseases [52]. More in-depth research is required to investigate the influence of ZEN on the homeostasis of Paneth cells, and consequently on the ISC microenvironment. In the small intestine, BD1 and BD3 are mostly expressed in enterocytes, and are constitutive in the case of BD1 and induced by inflammation in the case of BD3 [44]. While ZEN induced the expression of pBD1, pBD3 was down-regulated. Other than differences in the antimicrobial and chemotactic function [44], both defensins appear to play a role in the promotion/inhibition of migration of cancer cells, and the direction of the regulation depends on the tissue concerned [53]. In the human intestine, BD3 inhibits migration [54]. Further research is needed to understand the effect of the observed ZEN-induced dysregulation of defensins in pig. 

In contrast to the pro-proliferating Wnt/β-catenin signaling, TGF-β maintains a state of growth equilibrium and promotes epithelial differentiation towards the tips of the villus [55]. TGF-β is an immunosuppressive cytokine that actively participates in the gut immune cell homeostasis through the regulation of the growth and functions of dendritic cells, T natural killer and B cells [56]. TGF-β is also a potent growth inhibitor of intestinal epithelial cells [56]. Our results suggest that TGF-β signaling is suppressed after 4 h of exposure. This could be explained by the known ERα-mediated suppression of TGF-β-induced activation by interaction with Smad proteins [24]. 

The ZEN-mediated induction of inflammation has already been studied in different tissues in immune cells [10,11], the spleen [57,58], the liver, [11] and the intestine [8,12,30,59]. We observed no signs of inflammation either at the gene expression level (interleukins 8, 17, 18, 22, TNF-α, interferon gamma, Lipocalin 2, S100A8, S100A12) or at the protein level (IL-17α, IL-8, serum amyloid A3 and alpha-acid glycoprotein alpha 1). Significant down-regulation of inflammatory cytokines has also been reported in the duodenum of pigs exposed to ZEN [12]. These results could be linked to the ZEN-induced activation of ERα signaling, which has anti-inflammatory effects [60,61].

We investigated the expression of PPARα, PPARγ as well as that of other genes linked with adipokines, a family of metabolic sensing proteins, of which some members have been found to be regulated by ZEN in the intestine [30,40]. No differences in the expression of PPARα, PPARγ, serum amyloid A, lipocalin 2 or adiponectin were found, whereas the mRNA levels of the adipokines receptors for adiponectin (AdipoR2), resistin (CAP1) and chemerin (CMKLR1) were significantly up-regulated. Adipokines are peptides that signal the functional status of adipose tissue to targets in different tissues, including the intestine. Their main roles are the regulation of inflammatory processes and energy balance, mainly glucose metabolism, as well as cell proliferation and differentiation, through the activation of ERK1/2, Akt, and/or PI3K signaling pathways [62]. Resistin can induce the secretion of mucins in the intestine [63]. Interestingly, we observed nearly significant up-regulation of mucin-1 in our conditions. The effect of ZEN on the systemic levels and local signaling of adipokines merits further investigation not only in castrated males, but also in pre-pubertal females and adults from both sexes, since other known effects of ZEN suggest modulation of energy balance. Indeed, the anabolic effects of ZEN are well known and can disturb glucose levels in vivo in exposed pre-pubertal gilts [2,64,65]. The importance of these findings is the possible connection between these endocrine-disturbing effects and the development of metabolic diseases [66], a link that has been poorly investigated in the case of ZEN.

## 4. Conclusions

Our results provide new knowledge of the toxicity of ZEN and confirm that exposure to ZEN can promote the development of severe intestinal pathologies. ZEN increase ER-alpha, activate Wnt/β-catenin signaling and repress TGF-β in the small intestine of castrated male pigs, inducing a pro-proliferative/pre-cancerous phenotype, characterized by over-expression of MCM3 and VIM. This suggests that ZEN disturbs the ISC microenvironment, promoting a pro-cancerous, proliferative state in the intestine that has been already described in the literature [12,28,29,30,59]. Our results also show that ZEN can modulate the energy sensing-related phenotype of intestinal tissues without inducing inflammation. More research including models of different sex and age is needed to understand the influence of ZEN on the immune-metabolic status of exposed individuals. 

## 5. Materials and Methods

### 5.1. Toxin

Purified zearalenone (ZEN) was purchased from Sigma (St Quentin Fallavier, France), and dissolved in dimethylsulfoxide (DMSO; Sigma, St Quentin Fallavier, France) to obtain a concentration of 20 mM, aliquoted and stored at −20 °C. 

### 5.2. Animals and Culture of Pig Jejunal Explants

Six castrated male, 5-week old piglets, were provided by a local farm (Gaec de Calvignac, St. Vincent d’Autejac, France). The experiment was conducted under the guidelines of the French Ministry of Agriculture for animal research. The Ethics Committee of Pharmacology-Toxicology of Toulouse-Midi-Pyrénées approved all animal experimentation procedures (Toxcométhique; N°: TOXCOM/0136/PP). The collection of jejunum tissue and the preparation of explant cultures was as described previously [67,68,69], the only modification being changing the culture medium to Williams phenol red-free (Sigma, St Quentin Fallavier, France) supplemented with 1% of insulin transferrin-selenium (Sigma, St Quentin Fallavier, France), 1% penicillin (Eurobio, Courtaboeuf, France), 1% alanine-glutamine (Sigma, St Quentin Fallavier, France), 0.5% gentamycin (Eurobio, Courtaboeuf, France) and 50 mL of glucose (Sigma, St Quentin Fallavier, France). Explants were exposed to ZEN (100 µM) or vehicle only (DMSO; Sigma, St Quentin Fallavier, France) and incubated in the same conditions for a period of 1 h or 4 h. After treatment, tissues were snap frozen in liquid nitrogen, and the culture medium containing proteins leaked from explants was recovered. All samples were stored at −80 °C until analysis.

### 5.3. RNA Extraction and Real-time Quantitative PCR (RT-qPCR)

The pig jejunal explants were homogenized using 2 mL plastic bead tubes (MT Biomedicals, Illkirch, France) in 1 mL of Extract-All reagent (Eurobio, Courtaboeuf, France) in a Precellys Evolution tissue homogenizer (Bertin Technologies, Montigny-le-Bretonneux, France). ARN isolation and RT-qPCR were performed as described elsewhere [70,71]. Data analysis was carried out using LinRegPCR freeware [72], and normalized against two reference genes, TATA-Box Binding Protein (TBP) and Hypoxanthine guanine phosphoribosyl transferase 1 (HPRT1). Primers are described in Supplementary Table S2.

### 5.4. Protein Extraction and Western Blot

Proteins from jejunal explants were extracted using 2 mL plastic bead tubes (MT Biomedicals, Illkirch, France) in 0.5 mL of radioimmunoprecipitation assay buffer (RIPA buffer) containing protease- and phosphatase inhibitors (Sigma, St Quentin Fallavier, France) in a Precellys Evolution tissue homogenizer (Bertin Technologies, Montigny-le-Bretonneux, France). Total protein content was quantified (Bicinchoninic acid (BCA) protein assay, Thermo Fisher Scientific, Waltham, MA, USA). Total protein extracts (20 µg) were separated in 12.5% or 15% acrylamide-bisacrylamide gels, and transferred onto nitrocellulose membranes. Blots were blocked in RotiBlock (Carl Roth GmbH, Karlsruhe, Germany) and incubated with the corresponding primary antibody at 4 °C overnight under agitation (Supplementary Table S3). Detection was achieved using appropriate species-specific fluorescent secondary antibodies (Biotium, Inc., Fremont, CA, USA). Total protein staining of the membrane with SyproRuby blot stain (Thermo Fisher Scientific, Waltham, MA, USA) prior to blocking served as a loading control in all cases. Images were obtained by scanning Chemidoc (SyproRuby-stained membranes; Bio-Rad) or a Li-Cor Odyssey Infrared Imager (Fluorescent immunoblots; Li-Cor Biosciences, Lincoln, NE, USA). All the images were digitalized and analyzed with Image Studio Lite Software v5.2 (Li-Cor Biosciences, Lincoln, NE, USA). For each lane, band intensity values were normalized onto the respective overall protein staining.

### 5.5. Gelatin Zymography

The explant culture medium was concentrated (approximately 10-fold) using centrifugal filter units (Amicon Ultra-2 mL Centrifugal Filters, Merck-Millipore, Darmstadt, Germany). Total protein content was quantified (BCA protein assay, Thermo Fisher Scientific, Waltham, MA, USA) according to the manufacturer’s instructions. Protein aliquots (10 µg) were separated in 7.5% acrylamide-bisacrylamide gels containing 0.3 mg/mL gelatin from porcine skin (Sigma, St Quentin Fallavier, France) under non-reducing, non-denaturing conditions. Gels were incubated (2 × 30 min) in 2.5% Triton X-100 (Sigma) for protein renaturation and then proteolytic reaction was allowed to take place in 50 mM Tris-HCl, 50 mM NaCl, 10 mM CaCl2, pH 7.5, for 20 h at 37 °C, followed by staining with colloidal Coomassie blue stain (Sigma, St Quentin Fallavier, France). Gelatinolytic activity was visible as clear zones on a blue background. Images were obtained using a Li-Cor Odyssey Infrared Imager (Li-Cor Biosciences, Lincoln, NE, USA) and analyzed with Image Studio Lite Software v5.2 (Li-Cor Biosciences, Lincoln, NE, USA).

### 5.6. Statistical Analysis

Statistical differences in gene expression and protein abundance were determined using paired t-tests using GraphPad Prism statistical software version 6 (GraphPad Software, San Diego, CA, USA). The significance level was set at *p* < 0.05.

## Supplementary Materials

The following are available online at https://www.mdpi.com/2072-6651/12/2/113/s1, Table S1: Results of the RT-qPCR of pig intestinal explants exposed for 1 h or 4 h to 100 µM ZEN, Table S2: List of primers used in this study, Table S3: List of primary antibodies used in this study
